# A Hyperspectral Image Classification Method Based on Multi-Discriminator Generative Adversarial Networks

**DOI:** 10.3390/s19153269

**Published:** 2019-07-25

**Authors:** Hongmin Gao, Dan Yao, Mingxia Wang, Chenming Li, Haiyun Liu, Zaijun Hua, Jiawei Wang

**Affiliations:** 1 College of Computer and Information, Hohai University, Nanjing 211100, China; 2 Nantong Ocean and Coastal Engineering Research Institute, Hohai University, Nantong 226300, China

**Keywords:** hyperspectral image classification, generative adversarial networks, Semi-supervised classification, multi-discriminator generative adversarial network

## Abstract

Hyperspectral remote sensing images (HSIs) have great research and application value. At present, deep learning has become an important method for studying image processing. The Generative Adversarial Network (GAN) model is a typical network of deep learning developed in recent years and the GAN model can also be used to classify HSIs. However, there are still some problems in the classification of HSIs. On the one hand, due to the existence of different objects with the same spectrum phenomenon, if only according to the original GAN model to generate samples from spectral samples, it will produce the wrong detailed characteristic information. On the other hand, the gradient disappears in the original GAN model and the scoring ability of a single discriminator limits the quality of the generated samples. In order to solve the above problems, we introduce the scoring mechanism of multi-discriminator collaboration and complete semi-supervised classification on three hyperspectral data sets. Compared with the original GAN model with a single discriminator, the adjusted criterion is more rigorous and accurate and the generated samples can show more accurate characteristics. Aiming at the pattern collapse and diversity deficiency of the original GAN generated by single discriminator, this paper proposes a multi-discriminator generative adversarial networks (MDGANs) and studies the influence of the number of discriminators on the classification results. The experimental results show that the introduction of multi-discriminator improves the judgment ability of the model, ensures the effect of generating samples, solves the problem of noise in generating spectral samples and can improve the classification effect of HSIs. At the same time, the number of discriminators has different effects on different data sets.

## 1. Introduction

Remote sensing technology is a kind of long-distance earth observation technology which was raised in the 1960s. As a new discipline, it has attracted wide attention and research because of its establishment on many subjects. The purpose of remote sensing image processing is to restore image and extract information. Remote sensing images in different periods record the geographic information of a region’s dynamic changes, reflecting the evolution of urban, lake and other ecosystems, so it has profound research significance. By the 1980s, the imaging spectrometer was introduced and a number of satellites with panchromatic, multispectral and hyperspectral sensors were launched in the United States and Germany, which greatly improved the physical means of remote sensing detection technology. Hyperspectral remote sensing, also known as imaging spectral remote sensing, its full name is ‘hyperspectral resolution remote sensing.’ Compared with ordinary remote sensing image, hyperspectral remote sensing images (HSIs) have more and more refined bands. Each pixel in the image corresponds to hundreds of spectral bands, which contains abundant spectral information. In addition, HSIs contain precise spatial geometric information which can reflect the spatial distribution and geometric relationship of objects and realizes the organic combination of spectral information and spatial geometric information. Therefore, compared with panchromatic and multispectral remote sensing images, hyperspectral data has incomparable advantages in ground object recognition and classification.

The traditional mathematical method and the SVM method have great limitations for processing HSIs with a large amount of data. Many morphological methods are applied to the classification of HSIs to obtain the feature of spatial structure information of HSIs. In addition, the context information method is also used to retrieve classification results through regularization in the post-processing stage. The multi-view method has not been widely used in the classification of HSIs, which has a great application prospect. Zhao et al. [1] developed both supervised algorithm (S-MVML-LA) and unsupervised algorithm (UMVML-LA). These methods can learn a more discriminative latent space that contain sufficient information on all the feature sets (or observations) by using very few within-class and between class neighbors. Xu et al. [2] presented a novel robust learning algorithm for recovering the latent intact representations for multi-view examples. Xie et al. [3] introduced a general framework of multi-modal distance metric learning based on multi-wing harmonium model. Yu et al. [4] proposed a hierarchical bilinear pooling approach to fuse multi-layer features for fine-grained recognition, which combines inter-layer interactions and discriminative feature learning in a mutually-reinforced way. Wang et al. [5] investigated several kernel based methods for multimodal information analysis and fusion. These multi-view methods provide new ideas on how to combine spatial features and spectral features in HSIs for classification. In the future, we can consider the combination of multi-view method and deep learning for classification of HSIs.

In recent years, the emergence of deep learning has provided possibilities for the processing of large-scale data sets. Depending on the strong learning ability of the deep network itself, the original image processing methods of denoising, dimensionality reduction and other problems have been greatly improved. The deep neural network has become an effective feature extraction and classification algorithm. Dewa et al. [6] studied the dimensionality reduction and classification effect of DBN in large data based on hyperspectral images. Pedram et al. [7] proposed a self-elevating CNN model and introduced the concept of jitter for the first time to solve the over-fitting phenomenon in remote sensing image processing. At the same time, the application of deep learning in hyperspectral image classification is following closely. The classification model of joint spatial and spectral dimension based on stacked self-encoding proposed by Chen and Lin et al. [8] is a relatively early study. Later, Zhao and Yang [9] proposed a two-channel deep convolution neural network. The two-channel of CNN extracts spectral and spatial features respectively and uses transfer learning to improve performance in the case of small samples. Mou and Ghamisi [10] first used the cyclic convolution network with gated units to represent spectral pixels as sequential data, thus realizing hyperspectral image classification. In 2014, with the emergence of Generative Adversarial Networks (GAN) [11], deep neural network has been developed completely. Later, Goodfellow, the founder of GAN, elaborated on the reasons for the instability of GAN training in detail in the literature [12] and gave some solutions through experimental comparison, so as to better train the network. Wasserstein GAN established by Arjovsky M et al. [13,14] on this basis theoretically solves the problem of gradient disappearance. On the other hand, Salimans and Odena [15] proposed a Generative Adversarial Network using feature matching method in 2016, which achieved good results in semi-supervised classification by relying only on a small amount of training data. The early GAN was mainly used for unsupervised classification. At present, the application of the Generative Adversarial Network in the semi-supervised classification of images is still in the initial stage of development. Nasim Souly et al. [16] proposed a semi-supervised GAN model for semantics segmentation, which generates a large amount of data and realizes automatic image semantic annotation through multiple classifiers. Zhao Liyi, Sun Quan and others [17,18] applied GAN to image restoration and achieved good results in the restoration of damaged images. Jin Weidong et al. [19] applied a double discriminator generation adversarial network to the anomaly detection of catenary and could identify abnormal objects such as bird’s nest.

The theory of generative adversarial network, which can imitate the random input variables to generate similar types of images according to the original images, comes from the idea of zero-sum game in game theory [20]. This semi-supervised classification method by expanding data sets is a brand-new model in the field of image classification, which has great research prospects. Based on this, this paper studies the hyperspectral image classification algorithm based on generative adversarial network. In the process of hyperspectral image classification, there are some problems: on the one hand, due to the existence of different objects with same spectrum phenomenon, if only generating samples from spectral samples, it will produce the wrong detailed characteristic information. On the other hand, the gradient disappears in the original GAN model and the scoring ability of a single discriminator will limit the quality of samples generated by the generator. At the same time, the original GAN of single discriminator has the problem of insufficient diversity of generated samples, which is also known as Mode Collapse. Specifically, the generator maps different input to roughly the same output, that is, it generates samples with little difference and cannot learn all the potential distribution of input samples. In order to solve these problems and improve the performance of the model, this paper introduces the scoring mechanism of multi-discriminator cooperative work and proposes a multi-discriminator generative adversarial networks (MDGANs), which is applied to three hyperspectral data sets to complete semi-supervised classification. The adjusted criteria are more rigorous and accurate than the original ones and the corresponding features of the generated samples are more real. The influence of the number of discriminators on the classification results is also studied in this paper. The experimental results show that the multi-discriminator generative adversarial networks can improve the classification effect of the model to some extent. 

The main contributions of this paper are as follows.

(1) The MDGANs in this paper uses semi-supervised classification. GAN is basically used in the field of unsupervised learning. We have made some changes on the basis of the original GAN structure, that is, add a layer of softmax at the top of the discriminator as the classifier. At this time, the output of the discriminator is $l_{1},l_{2},\ldots l_{n}$, which corresponds to the label category. At this time, GAN can classify labeled raw data and unlabeled generated samples at the same time. Compared with the original data, the training samples are greatly increased. Therefore, semi-supervised GAN can be applied to the case of small samples to improve the accuracy of classification of small samples.
(2) We introduce the scoring mechanism of multi-discriminator cooperative work and on the basis of this propose a multi-discriminator generative adversarial networks (MDGANs). Compared with the original single discriminator GAN, MDGANs has more rigorous and accurate judgment conditions. The generated samples can show more real features. It can effectively solve the pattern collapse and diversity deficiencies of the original single discriminator GAN and solve the problem of noise signal in the generated spectral samples. We also study the general process of semi-supervised classification of hyperspectral images based on MDGANs. The specific process is shown in the third part of this paper.
(3) We study the influence of the number of discriminators on the classification results. The number of discriminators can affect the judgment ability of the whole model and has a great impact on the classification accuracy. The experimental results show that Indian Pines data set is most sensitive to the number of discriminators, Pavia University data set is least sensitive to the number of discriminators, MDGANs has different optimal number of discriminators in different data sets classification. For Indian Pines and Salinas data sets, when the number of discriminators is 5 can get ideal classification results and for Pavia University data set, choose three or more than three discriminators ideal classification results can be obtained.

The rest of this paper is organized as follows. Section 2 discusses the related research work. Section 3 describes the proposed method in detail. Section 4 presents comprehensive experimental results on three publicly available data sets. Section 5 concludes this paper. 

## 2. Related Work

Generative adversarial network is a brand-new model that combines generation model with discriminant model. The generation model learns the distribution of the original data and the discriminant model is used to determine whether the generated results are consistent with the original distribution. After adjusting its learning parameters, the original distribution can be best fitted. The birth of generative adversarial network provides a new unsupervised learning method for feature extraction. In the whole model, the samples are constantly trained against each other and the generator parameters are updated from the gradient feedback of the discriminator, which is independent of the distribution of the input data samples. In theory, the model is more universal. 

Typical generative models are Autoregressive Model and Variational Autoencoder (VAE), both of which are based on maximum likelihood. The autoregressive model is similar to Markov chain and belongs to the category of sequence generation. It operates on the image at the pixel level [21]. Variational autoencoder is a probability graph model, which usually includes two parts: encoding and decoding. It mainly constrains the encoding process and forces the decoder to generate reconstructed images [22]. The characteristic of generative adversarial network is that it does not need to define the distribution function directly and it relies on the initial noise information to fit and generate data. Compared with other generative models, GAN has the following advantages: (1) generating sample data in parallel without changing the boundary conditions; (2) generating function does not have too many restrictions; (3) Compared with VAE, GAN produces better image quality. However, the disadvantage of GAN as a generation model is that the training process of generator is not stable enough and it will be unable to train [11].

### 2.1. Structure and Application of Traditional GAN Model 

After introducing the basic principles of the discriminant model and the generation model, the next is to expand in detail how the optimization functions of the two models in GAN are solved and eventually how to reach the equilibrium state. 

Suppose $x=\{x_{1},x_{2}$,⋯$x_{m}\}$ represents the set of m real samples, z represents the random noise vector and $P_{\mathrm{data}}\left(x\right)$ represents the sample distribution of real data. $\{x_{1},x_{2}$,⋯$x_{m}\}$ is obtained from m samples sampled from $P_{\mathrm{data}}\left(x\right)$ and m noise samples from prior distribution $P_{\mathrm{prior}}\left(z\right)$ are recorded as $P_{z}\left(z\right)$. 

**Figure 1:**
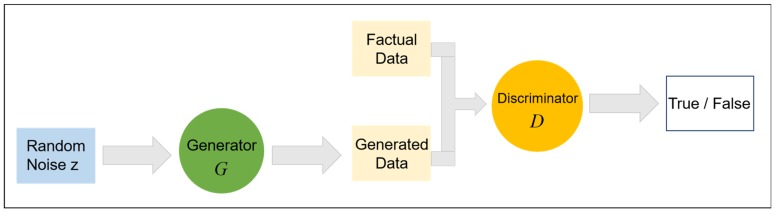
Basic Framework Structural Diagram of Generative Adversarial Network (GAN).

As shown in Figure 1, GAN has two main components in structure: one is generating network G, which receives random noise samples z and outputs a set of generated pictures, which are recorded as G(z); the other is discriminant network D, which judges the parameter x from generator or real data and outputs x as the probability of real data, which is recorded as D(x). D is equivalent to a two-classifier. According to the decision probability, the data can be divided into two categories: true and false. On the other hand, D can feed back the difference between the two to generator G through the expression of distance similarly, so that it can fit the real data as much as possible.

Thus, the objective function V(D,G) defined by the model can be expressed as [11]:

(1) $$\underset{G}{\min}\;\underset{D}{\max}V\left(D,G\right)=E_{x\sim P_{\mathrm{data}}\left(x\right)}\left[\mathrm{logD}\left(x\right)\right]+E_{z\sim P_{z}\left(z\right)}\left[\log\left(1-D\left(G\left(z\right)\right)\right)\right]$$

where E(*) denotes the expected value of the distribution function, D(*) denotes the probability of estimating the input sample from the real sample, z denotes the random noise samples and G(z) denotes the pseudo-samples. We can see that this is a minimax problem. In the case of given G, we first maximize V (D, G) and take D, then fix D and minimize V (D, G) to get G. Where, given G, maximized V(D,G) evaluates the difference or distance between the generated sample distribution function and the real sample distribution function.

In order to quantitatively describe the difference value, Jensen-Shannon distance (J-S divergence) is introduced to calculate the distance between two probability distributions. At this time, the GAN objective function is obtained by solving the following formula [11]:

(2) $$\underset{G}{\min}\;\underset{D}{\max}V\left(D,G\right)=-2\log2+2\mathrm{JSD}(P_{\mathrm{data}}\left(x\right)||P_{G}\left(x\right))$$

where $P_{\mathrm{data}}\left(x\right)$ denotes the distribution of real samples, $P_{G}\left(x\right)$ denotes the distribution of generated samples and JSD (*) denotes the calculation formula of J-S divergence. In fact, it can be proved that the GAN model can converge to special points and the discriminator D and generator G can obtain the optimal solution accordingly. In many cases, the solution process is called the game process of two models and the ideal result may reach the state of Nash Equilibrium.

But sometimes the game result in the GAN model cannot lead to the ideal result, that is, the gradient disappears, which is caused by the distance measurement method defined by J-S divergence. J-S divergence can be obtained from K-L divergence [23]. For the same random variable x, there are two separate probability distributions $P_{1},P_{2}$. We can use K-L divergence to measure the difference between the two distributions [24]:

(3) $$D_{\mathrm{KL}}(P_{1}||P_{2})=E_{x\sim P_{1}}\log\frac{P_{1}}{P_{2}}=E_{x\sim P_{1}}\left[{\mathrm{logP}}_{1}-{\mathrm{logP}}_{2}\right]$$

Due to the asymmetric property of K-L divergence, that is, $D_{\mathrm{KL}}(P_{1}||P_{2})$≠$D_{\mathrm{KL}}(P_{2}||P_{1})$, there exists meaningless value of K-L divergence. At this time, $P_{1},P_{2}$ are inconsistent.

Further, assuming that there are two distributions $P_{1}$ and $P_{2}$ and the average distribution of the two distributions is $M=\frac{P_{1}+P_{2}}{2}$, the J-S divergence between the two distributions can be expressed as the K-L divergence between $P_{1}$ and M plus the K-L divergence between $P_{2}$ and M divided by 2, that is [24]:

(4) $$D_{\mathrm{JS}}(P_{1}|{|P}_{2})=\frac{1}{2}\mathrm{KL}\left(P_{1}||\frac{P_{1}+P_{2}}{2}\right)+\frac{1}{2}\mathrm{KL}\left(P_{2}||\frac{P_{1}+P_{2}}{2}\right)$$

The range of J-S divergence of any two distributions is 0–log(n) and the maximum log(n) is obtained when the two distributions are far apart and do not overlap at all. log(n) is a constant and the gradient calculated at this point is undoubtedly 0. Therefore, the GAN model represented by J-S divergence has the phenomenon of gradient disappearance.

Although there are some shortcomings, it still does not affect GAN to play its role in many fields. In practical applications, GAN is mostly used in image field. As a generation model, the advantages of GAN are mainly embodied in avoiding the Markov chain learning mechanism, integrating various loss functions and GAN can still play its own advantages in scenarios where probability density cannot be calculated. For example, in the field of natural language processing, natural sentences can be realized by combining with RNN, such as the generation of poems [25]. It is more widely used in the field of image. From super resolution [26] to image restoration [27] and the emergence of facial attribute operation [28] in recent years, the application scene of generative adversarial network is constantly expanded and subdivided. In addition, GAN can combine with reinforcement learning. By introducing an unstable punishment-reward mechanism, the existence of adversarial network can promote more high-quality dialogues within the model.

Such applications undoubtedly open up a lot of areas in image processing that have not been involved before. On the other hand, GAN model is constantly improved and optimized.

### 2.2. GAN Model for Semi-Supervised Classification

The common semi-supervised learning methods include: self-training method, generation model, semi-supervised support vector machine (S3VMs), graph-based algorithm, multi-view algorithm and so on [29]. The graph-based algorithm maps the data set to a graph and the learning process corresponds to the data node spreading or propagating on the graph. Due to the fact that the solving process is matrix operation, the processing capacity of large-scale data sets is insufficient and the addition of new samples requires the reconstruction of graphs for training, so the applicability of this method is narrow. S3VMs needs to attach category balance as a constraint condition and the objective function is non-convex and difficult to calculate, the main research direction is to seek efficient optimization strategy. The multi-view algorithm requires samples to provide the set of attributes under other views and its applicability is also narrow. However, because the generated model can generate a large number of unlabeled sample data according to random variables, it provides a large number of data for model training to do feature extraction. If these unlabeled data are effectively used, it will undoubtedly improve the performance of the classification model. Among them, GAN is a widely used generation model.

**Figure 2:**
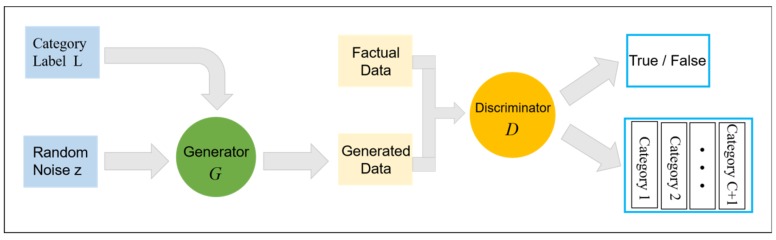
GAN Structure Diagram of Semi-supervised Classification.

GAN is basically used in the field of unsupervised learning after it was proposed but it was not found that there is research value in semi-supervised learning until later. GAN is a semi-supervised learning method when a small number of tags and multi-classifiers are added to it. However, the output of the original discriminator is true or false (0 or 1), which is a binary classification problem. In order to apply GAN to semi-supervised classification and realize hyperspectral image classification, we have made some changes based on the original GAN structure, that is, adding a layer of softmax to the top of the discriminator as the classifier. At this time, the discriminator output is $l_{1},l_{2},\ldots l_{n}$, which corresponds to the label category. $\mathrm{softmax}\left(x_{i}\right)=\frac{\exp\left(x_{i}\right)}{\sum_{j=1}^{n}\exp\left(x_{j}\right)}$ is a generalized form of Logistic, which is modeled by polynomial distribution, so it can combine different types of classifiers together to form multiple classifiers. Assuming that the original sample has many categories, the number of categories is counted as c. The samples generated by the generator are classified into category c + 1, so when training semi-supervised GAN model, softmax classifier also adds an output neuron, which is used to represent the probability that the discriminator model determines the input is false, namely, category c + 1. It can be seen that the GAN model can classify the labeled original data and the unlabeled generated samples at the same time and the training samples are much larger than the original data. Therefore, semi-supervised GAN can be applied to the case of small samples to improve the accuracy of classification algorithm. The specific semi-supervised GAN structure is shown in Figure 2, in which the category label L is added to the input to match the classification results at the output.

Because the output of the discriminator is no longer the probability of judging true or false, the loss function is different at this time. Semi-supervised GAN loss function has two parts, one is supervised learning loss function, the other is unsupervised loss function. The final loss function is obtained by adding the two functions together [12].

(5) $$L_{D}=-E_{x,y\sim p_{\mathrm{data}\left(x,y\right)}}\left[{\mathrm{logP}}_{\mathrm{model}}\left(y|x\right)\right]-E_{x\sim G}\left[{\mathrm{logP}}_{\mathrm{model}}\left(y=c+1|x\right)\right]$$

(6) $$\mathrm{Among},\;L_{\sup}=-E_{x,y\sim p_{\mathrm{data}\left(x,y\right)}}\left[{\mathrm{logP}}_{\mathrm{model}}\left(y|x,y<c+1\right)\right]$$

(7) $$\begin{array}{cc} L_{\mathrm{unsup}}= & -E_{x,y\sim p_{\mathrm{data}\left(x,y\right)}}\left[\log[1-P_{\mathrm{model}}\left(y=c+1|x\right)\right]\\ & -E_{x\sim G}\left[{\mathrm{logP}}_{\mathrm{model}}\left(y=c+1|x\right)\right] \end{array}$$

Let $D\left(x\right)=1-P_{\mathrm{model}}(y=c+1|x)$, the loss function of unsupervised learning can be simplified as follows:

(8) $$L_{\mathrm{unsup}}=-E_{x,y\sim p_{\mathrm{data}\left(x,y\right)}}\left[\mathrm{logD}\left(x\right)\right]-E_{z\sim \mathrm{noise}}\log\left[1-D\left(G\left(z\right)\right)\right]$$

where c is the number of categories, $p_{\mathrm{model}}(y|x)$ is the data distribution of each category and $p_{\mathrm{model}}(y=c+1|x)$ represents the probability of being false. It can be seen that the loss function $L_{\mathrm{unsup}}$ of unsupervised learning can actually be expressed as the loss function of GAN in formula (1). In the training process, for labeled samples, the cross-entropy loss is calculated, while for unlabeled samples, the two loss functions need to be minimized simultaneously.

Semi-supervised learning method can expand the data set, improve the generalization ability of the model through a large number of unlabeled data sets and learn the hidden features in unlabeled samples. It is suitable for scenarios where labeled data is missing. Before GAN was not used in the semi-supervised field, these unlabeled data were basically real data available. After the appearance of semi-supervised GAN, these unlabeled data can be synthesized manually, which solves some problems that cannot be handled because of the small number of original samples. Generative adversarial network can be used not only in image and speech generation but also in other image classification areas where depth model is good at. This is the basis of hyperspectral image classification in this paper.

## 3. Proposed Methods

### 3.1. Algorithmic Framework

**Figure 3:**
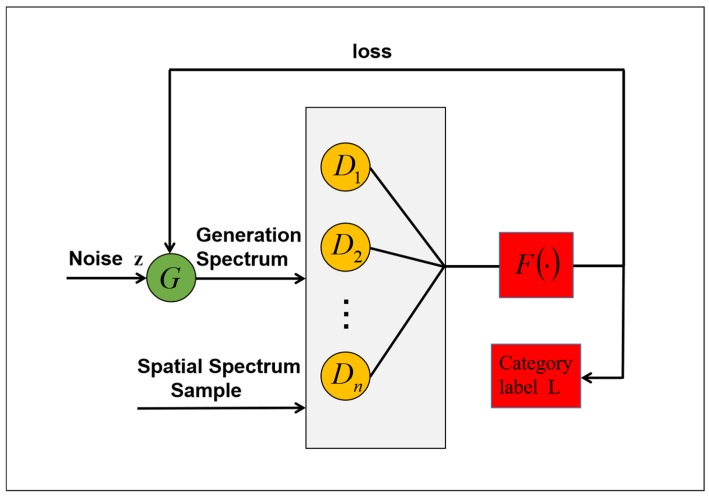
Structural Diagram of Multi-Discriminator Generative Adversarial Networks (MDGANs).

The structure of multi-discriminator generative adversarial network is shown in Figure 3. MDGANs consists of multiple discriminators *D*_1_, *D*_2_,…*D*_n_ and generators. Softmax classifier is used in the output layer of discriminator and the output of the discriminator corresponds to the category L of the label. Softmax is a generalized form of Logistic, which uses polynomial distribution as the model for modeling, so it can combine different types of classifiers together to form multiple classifiers. The noise z is input into the generator and the generation spectrum from the generator is input into the multi-discriminator together with the spatial spectrum sample of the data sets and the classification result is obtained, and the loss is returned to the generator. When training the MDGANs model, softmax classifier added an output neuron, which was used to represent the probability of the discriminator model judging that the input was false. It can be seen that the MDGANs model can classify both the original data with labels and the generated samples without labels and the training samples are greatly increased compared with the original data. The whole model is implemented on the Pytorch platform and (n − 1) discriminators are added. These discriminators jointly obtain the discriminating probability of a category and guide the generation of samples. In order to integrate the results of multiple discriminators, we average the results. There are three commonly used averaging methods: arithmetic averaging, geometric averaging and harmonic averaging. Finally, add softmax as classifier at the top of the multi-discriminator and output the category of the sample.

In many cases, the reason the generated samples are not good enough is not the inadequacy of the generator’s imitation ability but the inadequacy of the discriminator’s ability to distinguish falsehoods, which results in some generated samples deceiving the discriminator. After the introduction of multiple discriminators, more severe judgment ability can be introduced to ensure the effect of generating samples. The problem of noise signal in the generated spectral samples is solved and the general process of semi-supervised classification of hyperspectral images based on MDGANs is studied.

The overall scheme and processing flow of the whole system are roughly as follows. The hyperspectral data sets Indian Pines, Pavia University and Salinas are preprocessed on the Pytorch deep learning platform and input into MDGANs. High-quality hyperspectral image images are generated in the training stage and the test samples are classified in the testing stage. A complete iteration training process includes the following operations: generator G takes noise variable z and category information L as input and each iteration training learns sample information of corresponding category. Discriminator D determines whether the input is the actual spectral information or the generated spectral information. Then, the trained generated spectrum is mixed with the real spectrum in a certain proportion as the input of the softmax classifier (the function F in Figure 3). Each training cycle epoch completes the update of network weight until the MDGANs is stable. Finally, the softmax classifier outputs the categories of objects belonging to each pixel in the test set image.

In the classification stage, the calculation method of MDGANs judging category probability is as follows. The first method is to use bagging idea. The generator generates samples according to the input random noise signal. The probability of true or false samples is obtained by majority voting among multiple discriminators. The parameters of the generating network are updated by back propagation. After training is stable, the generated samples and real samples are classified by classifier to get the classification results of spectral data. The second integration method refers to Boosting’s algorithm flow. The weighted average of multiple discriminators is based on their own weights to get the classification results. At the same time, the weight index of each discriminator is updated according to the error function in the training stage. Considering that the second method is cumbersome, and the effect may not be ideal, the bagging method with better generalization ability is selected in the experiment. The specific training steps are as follows:

Step 1: Input the real spectrum sample as the original data set D;

Step 2: Random sampling is performed on D for several times to obtain the sampling set T;

Step 3: k discriminators are obtained after k times of put back sampling;

Step 4: Using majority voting between multiple discriminators to determine the generated samples;

Step 5: Update generator parameters and train discriminators until training is stable, then use softmax classifier to classify samples;

### 3.2. Classification of Spatial-Spectral Dimensions

**Figure 4:**
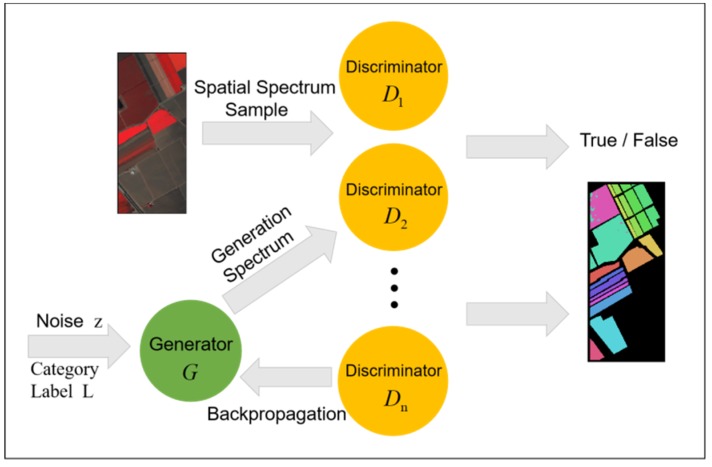
Semi-supervised Classification Process Flow Chart Based on MDGANs.

The phenomenon of different objects with same spectrum causes most classifiers to misclassify sample data. Therefore, the method of extracting spectral samples directly from hyperspectral images cannot achieve the best classification effect. Considering the spatial similarity of the same kind of objects, we can use the spatial texture features of hyperspectral images to extract the complete hyperspectral information by combining spectral samples and spatial samples and improve the accuracy of the classification algorithm. In order to extract spatial dimension information through GAN, we need real spatial samples and then we can get spatial-spectral samples by splicing them with spectral samples. We put this part of the work in the pretreatment part to complete, get the spatial-spectral samples and then submit them to the MDGANs to complete the classification task. Multiple discriminators jointly determine the generated spatial-spectral samples. After multiple rounds of training, when the MDGANs cannot determine the probability of true or false for samples, the training ends and the classification results are obtained. The MDGANs uses the softmax to calculate the probability that each sample in the sample set belongs to each category and the maximum probability value belongs to the category as the final classification result. The specific process flow of classification method is shown in Figure 4.

The acquisition of spatial-spectral samples can be divided into two ways. One is to use morphological profiling method [30] to obtain spatial features and then connect them with spectral features simply. Considering that the data is still high-dimensional at this time, dimensionality reduction is usually carried out before classification. Another way is to extract spectral samples with spatial information directly, such as 3D-Gabor filter or 2D-CNN, which can extract the whole spatial-spectral information and classify it. The feature fusion method adopted in this paper is a simple splicing method. Before this, hyperspectral images are preprocessed and the pre-processing stage includes normalization, standardization and zero-mean processing of hyperspectral data. Because there is a great possibility that a small number of pixels in hyperspectral images belong to the same category, when extracting spatial features, we use sliding window to extract the features of each band in turn with fixed-size windows. The extracted neighborhood blocks are represented as spatial samples and then connected with spectral features to generate new spatial samples in MDGANs which used for training. Compared with the original spectral samples, the noise information of the spatial-spectral samples after pretreatment is less, which is conducive to improving the quality of the generated samples and the final classification result.

### 3.3. Introduction of Dropout and Parameter Settings

Dropout, which is widely used in CNN to prevent model over-fitting, can also be used in the integration of multiple discriminators [31]. A robust scoring system generally takes into account the impact of outliers (such as the lowest and highest scores) and removes them. To this end, such a mechanism was introduced into the training process of MDGANs, that is, part of discriminators were filtered within each training cycle and the remaining discriminators participated in the voting score. In this way, in each training cycle, we dynamically integrate discriminators to guide the generator to generate spectral samples, so that MDGANs can learn a series of pattern features and avoid the phenomenon of pattern collapse in the training process.

Since the discriminator in the model is strongly classified by the integrated method, the network complexity of a single discriminator is appropriately reduced to prevent the learning speed of the generator from matching the training speed of the discriminator. In addition, in order to make the model more generalized and comparative, the classifier we added after the discriminator did not choose CNN but the general softmax multi-classifier, so as to better compare with the original GAN and other classical classification algorithms.

In the specific experiment, dropout = 0.5 is a better choice, that is, half of the total number of discriminators are randomly used in each training to participate in the voting score. In general, the scoring method of multi-discriminator makes the discriminator superior to the generator, so we update the multi-discriminator network parameters after the generator parameters are updated several times. For training convenience, the remaining initial super parameters include training rounds:epoch = 100, learning rate:lr = 0.001, batch_size = 256, dimension 30 of initial noise Z, number of discriminators:n = 5, mixing ratio of generated samples and real samples is 0.1 and it is set as semi-supervised learning.

## 4. Experimental Results

### 4.1. Experimental Configuration

All programs are implemented on the Pytorch Deep Learning Platform by Python Language and run on 8 GB memory and a 64-b Windows 7 OS desktop. Because the training samples of each experiment are randomly selected from the original data set, the results of each experiment are slightly different. In order to control the influence of different variables, all the experimental data in the following tables are the results of experiments under the same conditions.

### 4.2. The Evaluation Index and Data Set Description

Three commonly used hyperspectral datasets, Indian Pines, Pavia University and Salinas, are used to carry out experiments. The following is an introduction to several indicators used to assess the accuracy of classification and the specific situation of the above data sets.

In this paper, three indexes, including Overall Accuracy (OA), Average Accuracy (AA), Class Accuracy (CA) and Kappa coefficient, were used to evaluate the classification performance of the model. Among them, the OA is equal to the percentage of the number of pixels correctly classified in the test set to the total number of samples in the whole test set and the AA is the average of the classification accuracy of each category. Kappa coefficient is another index for evaluating classification accuracy. It is used to evaluate the consistency between classification results and real markers. The Kappa value greater than 0.8 means that the consistency between classification images and real information of objects is very high or the accuracy is very high. 0.61–0.80 means good consistency, 0.4–0.6 means medium consistency and less than 0.4 means poor consistency. Assuming that the ground objects to be classified in hyperspectral data set are of class C and that the sample number of the ground objects of class i being correctly classified into class i is $n_{ii}$ and the sample number of the ground objects of class i being wrongly classified into class j is *n_ij_*, then OA, AA, *CA_i_* (accuracy of class i-th) and Kappa coefficients are defined as follows:

(9) $$OA=\frac{\sum_{i=1}^{C}n_{ii}}{\sum_{i,j=1}^{C}n_{ij}}$$

(10) $$AA=\frac{\sum_{i=1}^{C}A_{i}}{C}$$

(11) $$CA_{i}=\frac{n_{ii}}{\sum_{j}^{C}n_{ij}}$$

(12) $$Kappa=\frac{OA-P_{e}}{1-P_{e}},among:P_{e}=\frac{1}{N^{2}}\sum_{i=1}^{C}a_{i}b_{i}$$

where, *A_i_* refers to the proportion of correctly classified samples of class i in the total samples of this class, N refers to the total samples to be classified, $a_{i}$ refers to the true samples of class i ground objects and *b_i_* refers to the predicted samples of class i ground objects, namely $a_{i}=\sum_{j}^{C}n_{ij}$, $b_{i}=\sum_{j}^{C}n_{ji}$.

**Figure 5:**
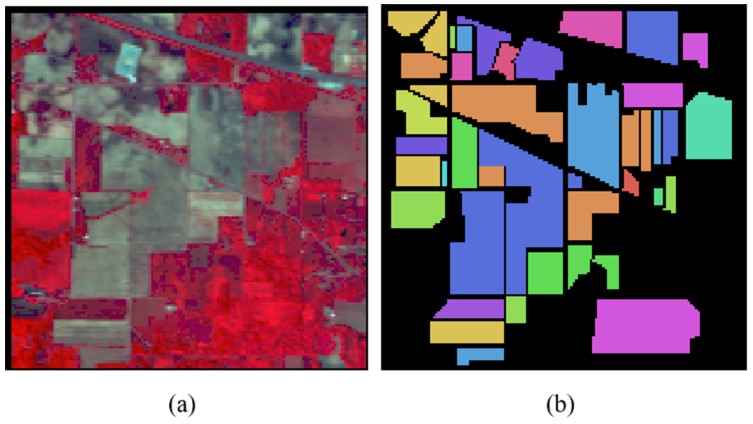
The Indian Pines Data Set. (**a**) False Color Images. (**b**) The Corresponding Ground Truth Maps.

Indian Pines: this hyperspectral image was completed in 1992 with an AVIRIS sensor and has 220 bands of 145 × 145, a spatial resolution of 20 m and a spectral range of 0.4 µm to 2.45 µm. The image area is the Northwest Indiana Agricultural Region. It is mainly used for agricultural research. It contains 16 types of ground objects, a total of 10,249 labeled pixels, of which 2/3 are crops, 1/3 are forests and other natural perennial plants. The number of samples of various types of ground objects is shown in Table 1. Figure 5 shows the false color image and the corresponding ground truth maps distribution of the hyperspectral image.

**Figure 6:**
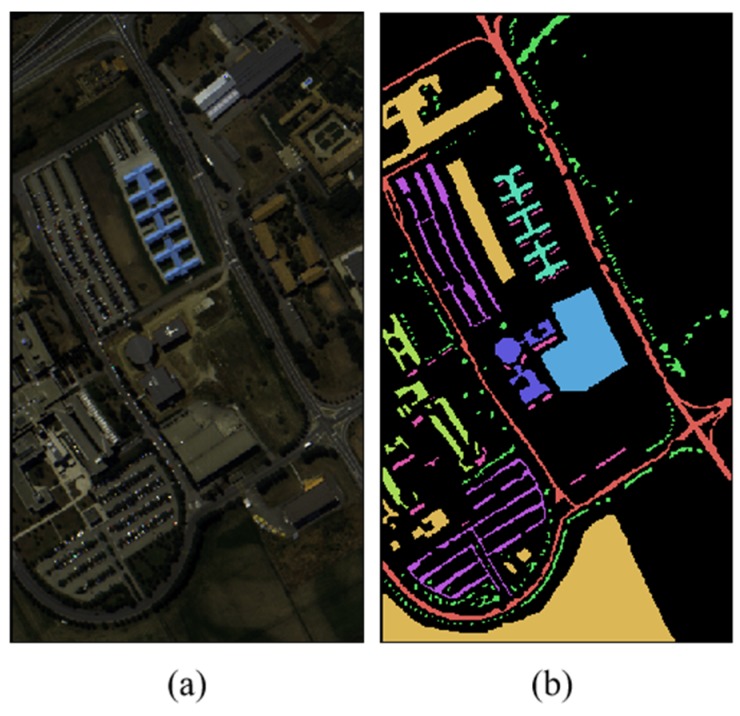
The Pavia University Data Set. (**a**) False Color Images. (**b**) The Corresponding Ground Truth Maps.

Pavia University: this hyperspectral image was taken by ROSIS sensor and the imaging area is Pavia University in northern Italy. Its spatial resolution is 1.3 m, the spectral band is 103, the size is 610 × 340 and it contains 9 categories of ground objects and 42,776 labeled pixels. The number of samples of various types of ground objects is shown in Table 2. Figure 6 shows the false color image and the corresponding ground truth maps distribution of the hyperspectral image.

**Figure 7:**
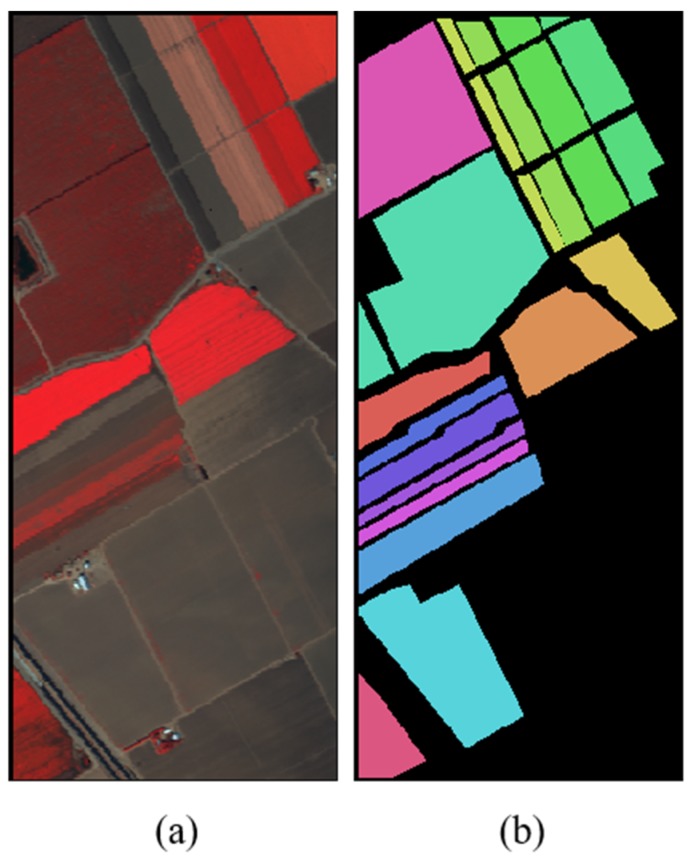
The Salinas Data Set. (**a**) False Color Images. (**b**) The Corresponding Ground Truth Maps.

Salinas: this hyperspectral image was collected by the AVIRIS sensor in the Salinas valley region of California. The image has a spatial resolution of 3.7 m, a size of 512 × 217 and contains 224 bands. Generally, 20 of the absorbent bands are removed and only 204 bands are reserved for related studies. The data set contains 16 categories of ground objects and a total of 54,129 labeled pixels. The number of samples of various types of ground objects is shown in Table 3. Figure 7 shows the false color image and the corresponding ground truth maps distribution of the hyperspectral image.

### 4.3. Performance Evaluation

**Figure 8:**
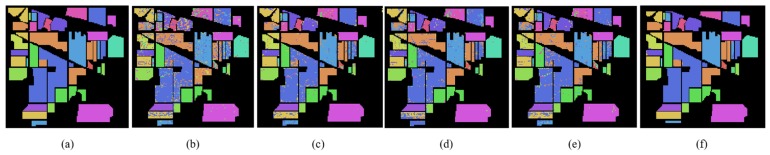
Classification Maps for the Indian Pines Data Set. (**a**) Ground-truth Classification Map; (**b**) KNN (73.74%); (**c**) NN (89.10%); (**d**) SVM (85.58%); (**e**) CNN (87.14%); (**f**) MDGANs (95.73%).

**Figure 9:**
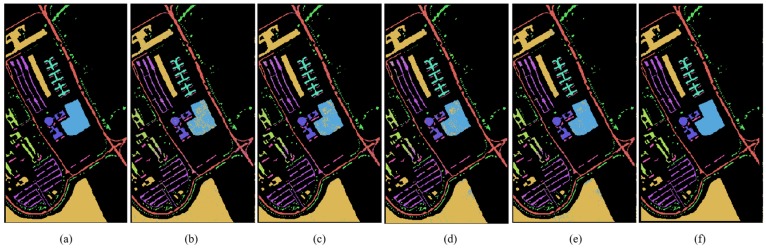
Classification Maps for the Pavia University Data Set. (**a**) Ground-truth Classification Map; (**b**) KNN (89.33%); (**c**) NN (93.86%); (**d**) SVM (94.37%); (**e**) CNN (94.79%); (**f**) MDGANs (94.67%).

**Figure 10:**
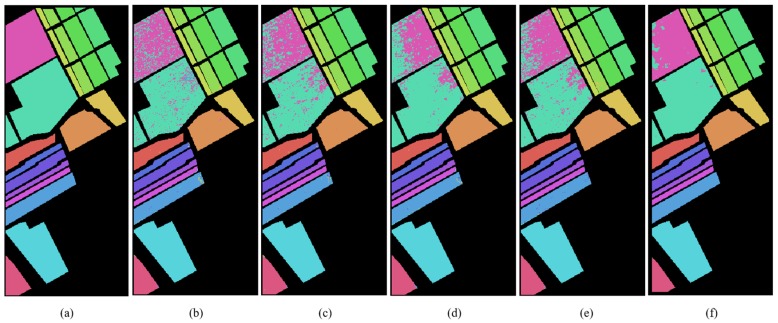
Classification Maps for the Salinas Data Set. (**a**) Ground-truth Classification Map; (**b**) KNN (91.90%); (**c**) NN (93.59%); (**d**) SVM (93.02%); (**e**) CNN (93.87%); (**f**) MDGANs (96.19%).

According to Table 1, Table 2 and Table 3, the Indian Pines, Pavia University and Salinas hyperspectral data sets of training set and test set are partitioned for semi-supervised classification. In order to evaluate the performance of MDGANs accurately, four models, K-Nearest Neighbor (KNN), Neural Network (NN), Support Vector Machine (SVM) and Convolutional Neural Networks (CNN), are selected as comparative experiments. Figure 8, Figure 9 and Figure 10 show the classification results of different methods. Table 4, Table 5 and Table 6 shows the classification accuracy of different methods in different types of objects. The most accurate value is marked in bold. 

Figure 8, Figure 9 and Figure 10 show the classification performance of different methods in three data sets: Indian Pines, Pavia University and Salinas. It can be clearly seen that the MDGANs has a better classification result in the classification of three different HSIs data sets.

Table 4, Table 5 and Table 6 shows the classification accuracy of different methods in three HSIs datasets. In Indian Pines dataset, the CA, OA, AA and Kappa of 16 ground objects are higher than those of the other four methods. In Pavia University dataset, the AA, Kappa and the CA of 8 ground objects are higher than those of the other four methods. The OA is 0.12% lower than CNN and the overall classification performance is still better than the other four methods. In Salinas dataset, the OA, AA, Kappa and the CA of 8 ground objects are higher than those of the other four methods.

As can be seen from Table 7, the Overall Accuracy of the Indian Pines data set, with a relatively low total data volume, has improved significantly as the proportion of training set increases from 5% to 50%. The Overall Accuracy of the Pavia University and Salinas data sets, with a relatively more total data volume, has improved not as obvious as that of Indian Pines dataset as the proportion of training set increases from 5% to 50%. The reason is that Pavia University and Salinas data set has more amount of data and the Overall Accuracy can still be more than 93% when only 5% training set are used. For Salinas data set, the 50% training set is not as effective as the 30% training set. The reason is that Salinas has the largest amount of data and overfitting occurs when the 50% training set is adopted. Therefore, it is not true that the higher the training set proportion is, the higher the classification accuracy will be.

From Table 8, we can see that for Indian Pines data set, the STMI-CSA proposed by Feng et al. and the CNN proposed by Romero et al. also adopt 30% training set, the MDGANs model proposed in this paper has certain advantages in OA, AA and Kappa. The DBN proposed by Chen et al. achieves high accuracy when using 50% training set. The MDGANs model proposed in this paper still has some advantages in OA, AA and Kappa. For Pavia University data set, the STMI-CSA proposed by Feng et al. adopt 30% training set, the MDGANs model proposed in this paper has certain advantages in OA, AA and Kappa. When using 60% training set, the SC-DBN proposed by Li et al. has 1.16% higher OA and 0.014 higher Kappa than the method proposed in this paper. The reason is that the training set is twice as large as the method in this paper, so the accuracy is slightly higher than the method in this paper. For Salinas data set, the STMI-CSA proposed by Feng et al. also adopt 30% training set, the MDGANs model proposed in this paper has certain advantages in OA, AA and Kappa.

Experimental results show that the MDGANs can introduce more severe judgment ability after introducing multiple discriminators, so as to ensure the quality of generating samples, solve the problem of noise signal in generated spectral samples and improve the classification effect of HSIs. Compared with some traditional classification methods, it has certain advantages.

**Figure 11:**
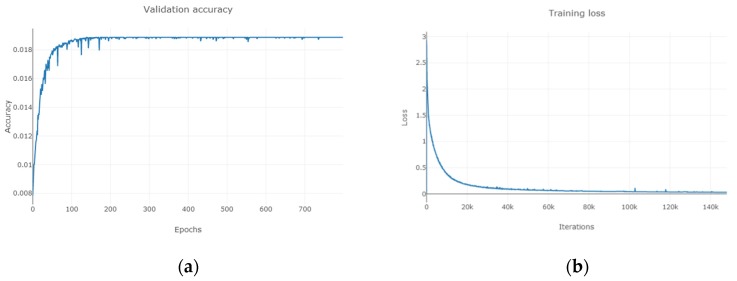
The Indian Pines. (**a**) The Change Curve of Accuracy-Training Period. (**b**) The Change Curve of Loss-Number of Iterations.

**Figure 12:**
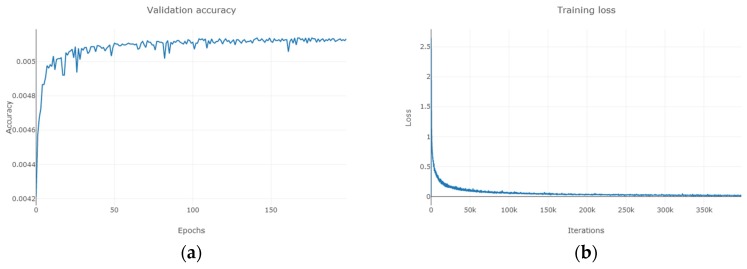
The Pavia University. (**a**) The Change Curve of Accuracy-Training Period. (**b**) The Change Curve of Loss-Number of Iterations.

**Figure 13:**
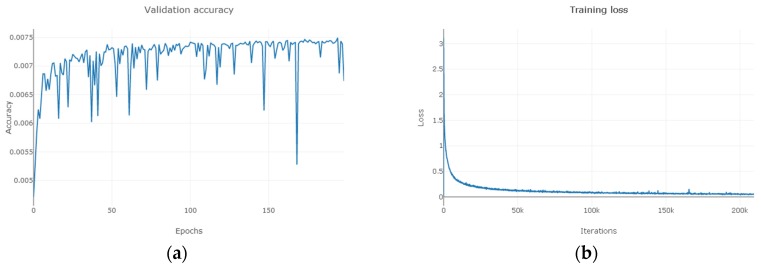
The Salinas. (**a**) The Change Curve of Accuracy-Training Period. (**b**) The Change Curve of Loss-Number of Iterations.

As can be seen from Figure 11, Figure 12 and Figure 13, for Indian Pines, the accuracy rate tends to stabilize when the training period reaches 150 and the loss function approaches 0 when the number of MDGANs iterations exceeds 80k and basically remains unchanged; for Pavia University, the accuracy rate tends to stabilize when the training period reaches 100 and the loss function approaches 0 when the number of MDGANs iterations exceeds 200k; for Salinas, the accuracy rate tends to stabilize when the training period reaches 70 and the loss function tends to stabilize 0 when the number of MDGANs iterations reaches 100k, because the data volume of Pavia University and Salinas is larger.

**Figure 14:**
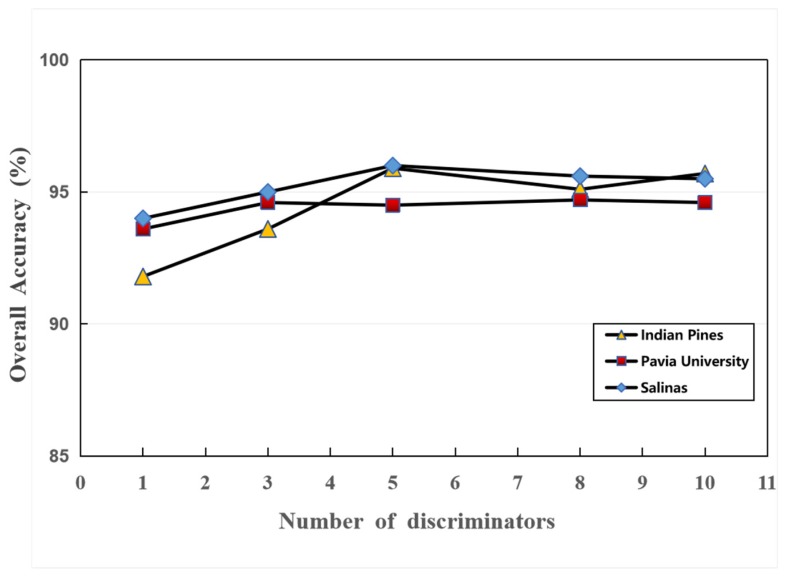
The Influence of Discriminator Number on Classification Accuracy on Different Data Sets.

In order to compare with the original GAN classification model, we studied the influence of the number of discriminators n on the classification results. In the experiment, control other variables remain unchanged and only the number of discriminators is changed, which is in order: 1, 3, 5, 8, 10, the learning rate is set to 0.001 and the training period is 800. Finally, the overall classification accuracy of MDGANs on Indian Pines, Pavia University and Salinas datasets is obtained. The experimental results are shown in Figure 14 as follows:

As can be seen from Figure 14, when n is 1, the MDGANs is GAN model of a single discriminator. Since the MDGANs in this paper does not combine CNN classifier to improve classification performance, the classification result of a single discriminator is inferior to SVM and CNN classification method. The method of ensemble learning can improve the generation effect of a single discriminator and the increase of the number of discriminators can enhance the judgement ability of the MDGANs and effectively improve the classification accuracy. The experimental results show that the method of multi-discriminator integration can improve the classification accuracy of the model to a certain extent. For the Indian Pines and Salinas data sets, the influence of the number of discriminators is obvious. When the number of discriminators is 5, a better classification result can be obtained and the running time of the MDGANs is not too long. However, for Pavia University data set, the number of discriminators does not have a significant impact and the selection of three or more discriminators can achieve ideal classification results. When the number of discriminators is 3, the running time of the MDGANs is relatively short.

A multi-discriminator ensemble method, the MDGANs, is applied to HSIs classification. By dynamically selecting discriminators to vote on the generated results, the phenomenon of pattern collapse in GAN is overcome and the quality of generated samples is guaranteed. At the same time, the MDGANs has the characteristics of diversity. Experiments show that the classification accuracy of this method is higher than SVM and CNN in three hyperspectral data sets. At the same time, we also studied the influence of the number of discriminators on the classification accuracy and the results showed that the classification result of the MDGANs was better than that of the single discriminator classification model.

## 5. Conclusions

In view of the fact that there are noise signals and too few training samples in hyperspectral images, this paper proposes Multi-Discriminator Generative Adversarial Networks applied to hyperspectral image classification -MDGANs. The model using the thought of ensemble learning applied to the optimization of structure of GAN, from hyperspectral image preprocessing to get spatial-spectral samples for training the generator. Then, the majority voting method is used to determine whether the generated samples are true or false and the voting score of multiple discriminators is used to guide the generation of samples. Compared with the original GAN, the stability of the training process is guaranteed, and the quality of the generated samples is improved. At the same time, the softmax classifier is used at the output to realize the multi-classification task of spectral samples. The experimental results show that the classification result of multi-discriminator network structure is better than that of single discriminator. At the same time, compared with CNN, SVM and some traditional methods, it has certain advantages in classification accuracy.

The MDGANs model proposed in this paper still has great room for improvement. Although the classification accuracy is improved, the training time also increases correspondingly. At the same time, there is still a lot of room for improvement in the means of extracting spatial features. How to better combine spectral features and spatial features is worthy of our deeper study. Multi-view learning has a wide range of applications in various fields. In the future, I will consider using multi-view learning to better integrate spectral features and spatial features and combine it with MDGANs model for HSIs classification, so as to achieve greater harvest.

## Figures and Tables

**Table 1:** Numbers of Training and Test Samples Used in The Indian Pines Data Set.

No.	Class	Training	Test
1	Alfalfa	14	32
2	Corn-notill	428	1000
3	Corn-mintill	249	581
4	Corn	71	166
5	Grass-pasture	145	338
6	Grass-trees	219	511
7	Grass-pasture-mowed	8	20
8	Hay-windrowed	143	335
9	Oats	6	14
10	Soybean-notill	292	680
11	Soybean-mintill	737	1718
12	Soybean-clean	178	415
13	Wheat	62	143
14	Woods	380	885
15	Buildings-Grass-Trees-Drives	116	270
16	Stone-Steel-Towers	28	65
Total		3076	7173

**Table 2:** Numbers of Training and Test Samples Used in the Pavia University Data Set.

No.	Class	Training	Test
1	Asphalt	1989	4642
2	Meadows	5595	13,054
3	Gravels	630	1469
4	Trees	919	2145
5	Painted metal sheets	404	941
6	Bare Soil	1509	3520
7	Bitumen	399	931
8	Self-Blocking Bricks	1105	2577
9	Shadows	284	663
Total		12,834	29,942

**Table 3:** Numbers of Training and Test Samples Used in the Salinas Data Set.

No.	Class	Training	Test
1	Brocoli_green_weeds_1	603	1406
2	Brocoli_green_weeds_2	1118	2608
3	Fallow	593	1383
4	Fallow_rough_plow	418	976
5	Fallow_smooth	803	1875
6	Stubble	1188	2771
7	Celery	1074	2505
8	Grapes_untrained	3381	7890
9	Soil_vinyard_develop	1861	4342
10	Corn_senesced_green_weeds	983	2295
11	Lettuce_romaine_4wk	320	748
12	Lettuce_romaine_5wk	578	1349
13	Lettuce_romaine_6wk	275	641
14	Lettuce_romaine_7wk	321	749
15	Vinyard_untrained	2180	5088
16	Vinyard_vertical_trellis	542	1265
Total		16,238	37,891

**Table 4:** Classification Results by Using Different Methods on the Indian Pines Data Set.

No.	Class	KNN	NN	SVM	CNN	MDGANs
1	Alfalfa	41.50%	78.00%	87.50%	64.20%	**95.20%**
2	Corn-notill	61.10%	88.10%	82.00%	84.60%	**99.20%**
3	Corn-mintill	59.60%	83.90%	77.90%	80.50%	**92.70%**
4	Corn	49.40%	78.50%	72.70%	73.00%	**97.20%**
5	Grass-pasture	86.20%	91.40%	92.90%	92.90%	**94.10%**
6	Grass-trees	90.20%	95.90%	95.10%	95.40%	**99.80%**
7	Grass-pasture-mowed	87.80%	88.90%	90.00%	87.20%	**97.60%**
8	Hay-windrowed	94.10%	97.60%	99.00%	97.50%	**99.90%**
9	Oats	48.00%	74.10%	33.30%	75.00%	**78.30%**
10	Soybean-notill	70.00%	86.00%	76.50%	84.60%	**96.70%**
11	Soybean-mintill	74.80%	88.80%	83.70%	86.10%	**97.90%**
12	Soybean-clean	51.90%	87.70%	87.40%	87.90%	**96.40%**
13	Wheat	91.90%	100.00%	97.60%	95.50%	**100.00%**
14	Woods	92.00%	94.70%	94.70%	93.90%	**99.40%**
15	Buildings-Grass-Trees-Drives	45.00%	69.90%	71.20%	65.40%	**81.60%**
16	Stone-Steel-Towers	91.80%	96.90%	94.60%	96.10%	**97.70%**
OA		73.74%	89.10%	85.58%	87.14%	**95.73%**
AA		70.96%	87.53%	83.51%	84.88%	**97.68%**
Kappa		0.700	0.876	0.835	0.853	**0.952**

**Table 5:** Classification Results by Using Different Methods on the Pavia University Data Set.

No.	Class	KNN	NN	SVM	CNN	MDGANs
1	Asphalt	91.50%	95.50%	95.10%	96.20%	**97.60%**
2	Meadows	93.50%	96.90%	**97.00%**	96.90%	95.20%
3	Gravels	73.60%	80.60%	83.50%	85.90%	**95.70%**
4	Trees	91.70%	96.10%	96.90%	96.90%	**99.00%**
5	Painted metal sheets	99.60%	99.60%	99.50%	99.60%	**99.90%**
6	Bare Soil	76.20%	93.50%	90.10%	92.70%	**99.90%**
7	Bitumen	81.70%	90.30%	88.50%	91.40%	**99.10%**
8	Self-Blocking Bricks	82.70%	83.80%	88.30%	89.70%	**98.70%**
9	Shadows	99.90%	99.90%	99.90%	99.90%	**99.90%**
OA		89.33%	93.86%	94.37%	**94.79%**	94.67%
AA		79.44%	93.01%	93.20%	94.36%	**98.50%**
Kappa		0.700	0.876	0.835	0.853	**0.931**

**Table 6:** Classification Results by Using Different Methods on the Salinas Data Set.

No.	Class	KNN	NN	SVM	CNN	MDGANs
1	Brocoli_green_weeds_1	99.40%	99.70%	**99.90%**	**99.90%**	97.30%
2	Brocoli_green_weeds_2	99.50%	99.60%	99.90%	99.80%	**100%**
3	Fallow	97.10%	99.30%	99.40%	97.20%	**99.80%**
4	Fallow_rough_plow	99.10%	99.40%	99.30%	99.30%	**99.50%**
5	Fallow_smooth	98.30%	99.30%	99.20%	97.60%	**100%**
6	Stubble	99.80%	**99.90%**	**99.90%**	**99.90%**	98.90%
7	Celery	99.40%	99.70%	**100%**	99.80%	99.40%
8	Grapes_untrained	83.50%	86.00%	85.20%	87.00%	**97.10%**
9	Soil_vinyard_develop	99.20%	99.70%	99.60%	99.40%	**99.70%**
10	Corn_senesced_green_weeds	93.20%	96.50%	96.90%	96.90%	**98.10%**
11	Lettuce_romaine_4wk	94.60%	96.60%	**98.30%**	97.80%	98.00%
12	Lettuce_romaine_5wk	98.30%	99.60%	99.60%	**99.70%**	98.10%
13	Lettuce_romaine_6wk	96.90%	99.50%	**99.90%**	98.90%	98.60%
14	Lettuce_romaine_7wk	93.90%	98.40%	**98.60%**	97.50%	98.00%
15	Vinyard_untrained	73.50%	77.90%	72.50%	79.00%	**93.90%**
16	Vinyard_vertical_trellis	98.90%	98.80%	99.40%	**99.50%**	88.90%
OA		91.90%	93.59%	93.02%	93.87%	**96.19%**
AA		95.29%	96.87%	96.73%	96.83%	**97.74%**
Kappa		0.910	0.929	0.922	0.932	**0.958**

**Table 7:** The Overall Accuracy of Three Data Set under Class Imbalance Condition.

Training Set	5%	10%	30%	50%
**Indian Pines**	85.12%	91.04%	95.73%	95.98%
**Pavia University**	93.51%	94.30%	94.67%	95.14%
**Salinas**	93.44%	94.85%	96.19%	96.03%

**Table 8:** Comparison with Other Methods.

Dataset	Reference	Method	Training Set	OA	AA	Kappa
Indian Pines	Feng et al. [32]	STMI-CSA	30%	83.70%	73.50%	0.813
	Romero et al. [33]	CNN	30%	-	-	0.840
	Chen et al. [34]	DBN	50%	91.34%	89.70%	0.901
	-	MDGANs	30%	95.73%	97.68%	0.952
Pavia University	Feng et al. [32]	STMI-CSA	30%	94.60%	92.80%	0.928
	Li et al. [35]	SC-DBN	60%	95.83%	94.67%	0.945
	-	MDGANs	30%	94.67%	98.50%	0.931
Salinas	Feng et al. [32]	STMI-CSA	30%	93.70%	96.80%	0.930
	-	MDGANs	30%	96.19%	97.74%	0.958

## References

[1] Zhao Y., You X., Yu S., Xu C., Yuan W., Jing X., Zhang T., Tao D. (2018). Multi-view manifold learning with locality alignment. Pattern Recognit..

[2] Xu C., Tao D., Xu C. (2015). Multi-View Intact Space Learning. IEEE Trans. Pattern Anal. Mach. Intell..

[3] Xie P., Xing E. Multi-Modal Distance Metric Learning. Proceedings of the International Joint Conference on Artificial Intelligence.

[4] Yu C., Zhao X., Zheng Q., Zhang P., You X. (2018). Hierarchical Bilinear Pooling for Fine-Grained Visual Recognition. arXiv.

[5] Wang Y., Guan L., Venetsanopoulos A.N. (2012). Kernel Cross-Modal Factor Analysis for Information Fusion with Application to Bimodal Emotion Recognition. IEEE Trans. Multimed..

[6] Arsa D.M.S., Jati G., Mantau A.J., Wasito I. Dimensionality reduction using deep belief network in big data case study: Hyperspectral image classification. Proceedings of the International Workshop on Big Data & Information Security.

[7] Ghamisi P., Chen Y., Zhu X.X. (2016). A Self-Improving Convolution Neural Network for the Classification of Hyperspectral Data. IEEE Geosci. Remote Sens. Lett..

[8] Chen Y., Lin Z., Zhao X., Wang G., Gu Y. (2014). Deep Learning-Based Classification of Hyperspectral Data. IEEE J. Sel. Top. Appl. Earth Obs. Remote Sens..

[9] Yang J., Zhao Y., Chan C.W., Yi C. Hyperspectral image classification using two-channel deep convolutional neural network. Proceedings of the 2016 IEEE International Geoscience and Remote Sensing Symposium (IGARSS).

[10] Mou L., Ghamisi P., Zhu X.X. (2017). Deep Recurrent Neural Networks for Hyperspectral Image Classification. IEEE Trans. Geosci. Remote Sens..

[11] Goodfellow I.J., Pouget-Abadie J., Mirza M., Xu B., Warde-Farley D., Ozair S., Courville A., Bengio Y. Generative Adversarial Nets. Proceedings of the International Conference on Neural Information Processing Systems.

[12] Salimans T., Goodfellow I., Zaremba W., Cheung V., Radford A., Chen X. (2016). Improved Techniques for Training GANs. arXiv.

[13] Arjovsky M., Chintala S., Bottou L. (2017). Wasserstein GAN. arXiv.

[14] Arjovsky M., Bottou L. (2017). Towards principled methods for training generative adversarial networks. arXiv.

[15] Odena A. (2016). Semi-Supervised Learning with Generative Adversarial Networks. arXiv.

[16] Souly N., Spampinato C., Shah M. Semi Supervised Semantic Segmentation Using Generative Adversarial Network. Proceedings of the 2017 IEEE International Conference on Computer Vision (ICCV).

[17] Zhao L. (2018). Research on Image Restoration Algorithm Based on Generative Confrontation Network. Master’s Thesis.

[18] Sun Q., Zeng X. (2018). Image restoration based on generated confrontation network. Comput. Sci..

[19] Jin W., Yang P., Tang P. (2018). Double discriminators generate antagonistic networks and their applications in OCS nest detection and semi-supervised learning. Chin. Sci. Inf. Sci..

[20] Behingya J.N. (1996). Non-cooperative Game. Syst. Eng..

[21] van den Oord A., Kalchbrenner N., Vinyals O., Espeholt L., Graves A., Kavukcuoglu K. (2016). Conditional Image Generation with PixelCNN Decoders. arXiv.

[22] Kingma D.P., Welling M. (2013). Auto-encoding variational bayes. arXiv.

[23] Tsai S.C., Tzeng W.G., Wu H.L. (2005). On the Jensen-Shannon Divergence and Variational Distance. IEEE Trans. Inf. Theory.

[24] Barz B., Rodner E., Garcia Y.G., Denzler J. (2018). Detecting Regions of Maximal Divergence for Spatio-Temporal Anomaly Detection. IEEE Trans. Pattern Anal. Mach. Intell..

[25] Rajeswar S., Subramanian S., Dutil F., Pal C., Courville A. (2017). Adversarial Generation of Natural Language. arXiv.

[26] Ledig C., Theis L., Huszar F., Caballero J., Cunningham A., Acosta A., Aitken A., Tejani A., Totz J., Wang Z. (2016). Photo-Realistic Single Image Super-Resolution Using a Generative Adversarial Network. arXiv.

[27] Yang C., Lu X., Lin Z., Shechtman E., Wang O., Li H. (2016). High-Resolution Image Inpainting using Multi-Scale Neural Patch Synthesis. arXiv.

[28] Liu M.Y., Tuzel O. (2016). Coupled Generative Adversarial Networks. arXiv.

[29] Liang J., Gao J. (2009). Research progress of semi-supervised learning. J. Shanxi Univ..

[30] Ghamisi P., Mura M.D., Benediktsson J.A. (2015). A Survey on Spectral-Spatial Classification Techniques Based on Attribute Profiles. IEEE Trans. Geosci. Remote Sens..

[31] Mordido G., Yang H., Meinel C. (2018). Dropout-GAN: Learning from a Dynamic Ensemble of Discriminators. arXiv.

[32] Feng J. (2014). Remote Sensing Image Classification Based on Soft Computing and Mutual Information Theory. Master’s Thesis.

[33] Romero A., Gatta C., Camps-Valls G. (2015). Unsupervised Deep Feature Extraction for Remote Sensing Image Classification. IEEE Trans. Geosci. Remote Sens..

[34] Chen Y., Zhao X., Jia X. (2015). Spectral-Spatial Classifification of Hyperspectral Data Based on Deep Belief Network. IEEE J. Sel. Top. Appl. Earth Obs. Remote Sens..

[35] Li C., Wang Y., Zhang X., Gao H., Yang Y., Wang J. (2019). Deep Belief Network for Spectral-Spatial Classification of Hyperspectral Remote Sensor Data. Sensors.

